# Effects of inpatient creatinine testing frequency on acute kidney injury identification and staging: a historical cohort study

**DOI:** 10.1007/s11096-023-01697-4

**Published:** 2024-02-05

**Authors:** Catarina Luz Oliveira, Filipa Duarte-Ramos, Filipa Alves da Costa, Fernando Fernandez-Llimos

**Affiliations:** 1 iMED, Research Institute for Medicines, Faculty of Pharmacy, Universidade de Lisboa, Lisbon, Portugal; 2 Hospital de Vila Franca de Xira, Vila Franca de Xira, Portugal; 3 EPIUnit-Instituto de Saúde Pública, Universidade do Porto, Porto, Portugal; 4 Laboratory of Pharmacology, –Applied Molecular Biosciences Unit, i4HB–Institute for Health and Bioeconomy, Faculty of Pharmacy, Universidade of Porto, Porto, Portugal

**Keywords:** Acute kidney injury, Drug-related side effects and adverse reactions, Hospital, Pharmacoepidemiology, Pharmacy service, Retrospective studies

## Abstract

**Background:**

Acute kidney injury (AKI) is a multifactorial condition often induced by drugs commonly used in hospitals. Identifying and staging AKI necessitates frequent monitoring of renal function.

**Aim:**

To assess the impact of real-world hospital practices regarding serum creatinine (SCr) testing on the identification and staging of AKI, and its implications for adjusting drug doses.

**Method:**

A historical cohort study utilizing hospital records from all adult patients admitted between 01/06/2018 and 31/12/2020 was conducted. Patients with no SCr assessment during their stay or those with an SCr at admission ≥ 2 mg/dL were excluded. AKI was determined using two criteria, namely AKIN and KDIGO, considering the time intervals between two SCr tests as outlined in the criteria. Additionally, patients with SCr increases exceeding AKI limits, regardless the time interval, were also identified. The estimated glomerular filtration rate (eGFR) and kinetic eGFR (KeGFR) were calculated.

**Results:**

During the study period, 17,269 hospitalizations and 62,255 SCr tests were recorded. Among the 17,032 hospitalizations with a length of stay > 48 h, 46.8% experienced periods with no SCr tests performed for more than 48 h. Any stage of AKI was identified in 7.0% of patients and in 9.1% using AKI and KDIGO criteria, respectively. Ignoring time limits in both criteria revealed potential AKI in 1942 patients (11.2%), indicating a potential underdiagnosis of AKI by 37.5% or 19.1%, depending on the criteria used. A total of 76 drugs requiring dose adjustment in patients with eGFR ≤ 50 ml/min were prescribed in 78.5% admissions. These drugs were prescribed in 87.9% of patients potentially underdiagnosed with AKIN and in 88.9% with KDIGO.

**Conclusion:**

There is a need for changes in the established hospital procedures to ensure more frequent testing of SCr levels. Implementing an advanced scope of practice for clinical pharmacists could support these changes.

**Supplementary Information:**

The online version contains supplementary material available at 10.1007/s11096-023-01697-4.

## Impact statements

- This real-world study highlights a substandard practice in assessing inpatient serum creatinine, impeding the timely identification of acute kidney injury occurrences.
- Patients not meeting AKI criteria due to delays in creatinine assessment might have developed AKI at various severity stages.
- Patients inadvertently developing AKI are prescribed medications for which doses should have been adjusted based on their renal function levels.
- Clinical pharmacists could play a crucial role in monitoring or ordering creatinine tests to discontinue nephrotoxic drugs and recommending dose adjustments in AKI patients.

## Introduction

Renal function is routinely assessed in clinical practice through various estimated glomerular filtration rate (eGFR) equations, incorporating serum creatinine (SCr) tests and other anthropometric factors such as age, weight, stature, and race. Studies have demonstrated that even a minimal increase in SCr can significantly influence morbidity, mortality, and associated hospital costs [1–3].

Acute Kidney Injury (AKI) is a prevalent and significant complication encountered by hospitalized patients. The incidence of AKI among inpatients exhibits considerable variability, with potential underdiagnosis ranging from over 20% in developed countries [4] to 7% in developing countries [5]. While multifactorial in nature, AKI can be triggered by exposure to nephrotoxic drugs [6] and other factors associated with hospitalization. Furthermore, it is widely recognized that the use of nephrotoxic medications increases the risk of progressing to chronic renal failure [7]. Identification and staging of AKI severity can be accomplished through various criteria, including the Risk, Injury, and Failure; and Loss, and End-stage (RIFLE) [8], the Acute Kidney Injury Network (AKIN) [9], or the Kidney Disease Improving Global Outcomes (KDIGO) [10].

Dose adjustments for renally excreted drugs are common practice in patients with chronic kidney disease (CKD) and undergoing renal replacement therapies. However, clinical practice guidelines lack clear recommendations on adjusting drug doses for patients with AKI. Assessing renal function, especially the impact of temporary renal impairment during AKI, poses challenges [11]. Traditional equations used for CKD may not accurately reflect renal drug clearance during AKI [12], and novel approaches based on different biomarkers (e.g., cystatin C) are not yet widely integrated into clinical routines [13]. Kinetic estimated glomerular filtration rate (KeGFR) has emerged as an alternative for estimating renal function in situations where SCr levels are changing rapidly, as observed in AKI [14].

Pharmacists have demonstrated their pivotal role in optimizing medication through drug dose adjustments in patients with CKD or undergoing renal replacement therapy [15]. Moreover, clinical pharmacists can significantly contribute to mitigating the toxic effects of AKI-inducing drugs and facilitating the necessary dose adjustments following AKI occurrences [16–18]. However, for precise identification of AKI onset, clinical pharmacists require timely access to inpatients' SCr levels, measured at least every 48 h when using AKIN criteria [9] or every 7 days for KDIGO or RIFLE criteria [8, 10]. Therefore, this study intended to investigate the extent to which clinical pharmacists in Portugal have timely access to the essential data to ensure patient safety by identifying AKI in hospitalized patients.

### Aim

This study aimed to assess the impact of real-world hospital practices regarding SCr testing on the identification and staging of AKI and its implications for drug dose adjustments.

### Ethics approval

The study received approval from the Ethics Committee of Hospital Vila Franca de Xira (Ref: 19.11.2020, approved 11.02.2021). According to Portuguese legislation, patients' signatures on informed consent forms are not required for the use of secondary data from medical records if the data are anonymized, and the study is approved by an ethics committee [19].

## Method

### Study design and participants

A historical cohort study was conducted using secondary data extracted from hospital medical records. The study included all adult patients (over 18 years old) admitted to Hospital Vila Franca de Xira in Portugal between June 1st, 2018, and December 31st, 2020. Patients were excluded if they had no SCr assessment during their stay or if a clear renal impairment was evident, demonstrated by an SCr at admission of 2 mg/dL (177 mmol/L) or higher.

Data were extracted from the hospital medical records (Glintt Clinical Solutions). Extracted files encompassed patients' demographic information (sex at birth, age), hospital details (admission and discharge dates, service), baseline parameters (weight, stature), prescribed medications during hospitalization, and results of SCr tests, including corresponding dates, obtained from the clinical laboratory system.

To ensure data anonymization, patients' identification and hospitalization numbers were transformed into two sequential series using an anonymization key. Access to the anonymization key was limited to the clinical staff at the hospital who had regular access to patients' medical records.

### Data analysis

The length of stay (LoS) was determined by adding 1 day to the difference between discharge and admission dates, then converted into hours by multiplying by 24. A list of drugs requiring dose adjustment when CrCl reaches 50 mL/min was obtained from the hospital’s source, The Renal Drug Handbook 2nd edition [20].

AKI was assessed using two criteria: Acute Kidney Injury Network (AKIN) [9] and Kidney Disease Improving Global Outcomes (KDIGO) [10], as outlined in Table 1. Initial identification and staging were performed by considering the specified time intervals for SCr increases in accordance with the criteria. Subsequently, a second identification and staging process was carried out without accounting for time intervals, considering the entire length of the patient's stay.

**Table 1:** Criteria used to identify and stage acute kidney injury

Criteria	Time interval	
	AKIN	KDIGO
Stage 1		
Increase in serum creatinine ≥ 1.5 × above baseline	48 h	7 days
Absolute increase in serum creatinine ≥ 0.3 mg/dL (26.4 mmol/L)	48 h	48 h
Stage 2		
Increase serum creatinine ≥ 2 × above baseline	48 h	7 days
Stage 3		
Absolute increase in serum creatinine ≥ 4 mg/dL (354 mmol/L)	48 h	7 days
Increase serum creatinine ≥ 3 × above baseline	48 h	7 days

AKIN: Acute Kidney Injury Network; KDIGO: Kidney Disease Improving Global Outcomes

Utilizing all recorded SCr values, the eGFR was calculated using the Chronic Kidney Disease Epidemiology Collaboration (CKD-EPI) creatinine-based equation [21]. Additionally, KeGFR was determined using the Chen equation [14]. For KeGFR, CKD-EPI served as the eGFR, and SCr at admission was considered the steady-state creatinine. The lowest value between these two renal function metrics was identified for each hospitalization.

Descriptive analyses were performed, presenting results as mean and standard deviation (SD) or median and interquartile range (IQR), depending on variable distribution. Normality was assessed using the Shapiro-Wilks test, supplemented by visual inspection of the quintile-quintile (Q-Q) plot. Non-parametric tests, including Spearman’s rho for correlation and Mann–Whitney and Kruskal–Wallis tests for assessing differences between two or more independent groups, were employed. Significance was set at *p* < 0.05.

## Results

During the study period, the hospital information system recorded 41,018 admissions. Of these, 7,478 were hospitalizations of paediatric patients. Among the 33,539 adults (excluding 1 with unknown age), 13,061 hospitalizations lacked SCr measurements, and 3220 presented with an SCr at admission exceeding 2 mg/dL (177 mmol/L). Consequently, 17,269 hospitalizations were included for analyses, representing 12,694 unique patients, with a mean age of 66.7 years (SD = 18.7), 52.6% were females, and a mean weight of 73.7 kg (SD = 15.9) among the 12,655 with recorded weights. Notably, 9779 patients were admitted only once during the study period, while 8 had more than 10 admissions (with a maximum of 15). The hospitalizations were distributed over the study years: 3901 in the first 6 months of 2018, 7139 in 2019, and 6229 in 2020. The median LoS was 9.0 days (IQR 6.0:13.0).

A total of 62,254 SCr tests were conducted. SCr levels at admission exhibited a median of 0.94 mg/dL (IQR 0.78:1.20) [83 mmol/L (IQR 69:106)], within the range of 0.18–1.99 mg/dL (non-normally distributed). Basal SCr positively correlated with age (Spearman’s rho = 0.356; *p* < 0.001) and was significantly higher in male patients (median = 1.00 mg/dL, IQR = 0.84:1.30) compared to females (median = 0.89 mg/dL, IQR = 0.70:1.16) (Mann–Whitney *p* < 0.001). Among the 17,032 hospitalizations with longer LoS than 48 h, 46.8% (8075) experienced time lags exceeding 48 h with no SCr tests performed. Among the 10,476 hospitalizations lasting longer than 7 days, 3.5% (601) had periods with no SCr tests longer than 7 days.

Any stage of acute kidney injury (AKI) was identified in 1,214 patients (7.0%), strictly following the AKIN criteria, and in 1572 patients (9.1%) using KDIGO criteria. When not considering time intervals in both criteria, potential AKI could have occurred in 1942 patients (11.2%). Differences in incidence, considering and ignoring time intervals, are detailed in Table 2. Overall, 728 patients not identified as AKI with the strict use of AKIN time intervals and 370 patients with KDIGO time intervals had SCr elevations exceeding the limits for both criteria. This represents a potential underdiagnosis of AKI by 37.5% and 19.1%, respectively. Detailed differences in potential AKI stages when not considering time intervals are provided in Supplementary file 1.

**Table 2:** Inpatients achieving acute kidney injury when considering or ignoring the time intervals of creatinine elevations

N = 17,269 hospitalizations	Ignoring time for SCr changes	AKIN time intervals	KDIGO time intervals
No AKI	15,327 (88.6%)	16,055 (93.0%)	15,697 (90.9%)
Stage 1	1608 (9.3%)	1042 (6.0%)	1260 (7.3%)
Stage 2	257 (1.5%)	140 (0.8%)	241 (1.4%)
Stage 3	77 (0.4%)	32 (0.2%)	71 (0.4%)

AKI: Acute Kidney Injury; AKIN: Acute Kidney Injury Network; KDIGO: Kidney Disease Improving Global Outcomes; SCr: Serum creatinine

**Fig. 1:**
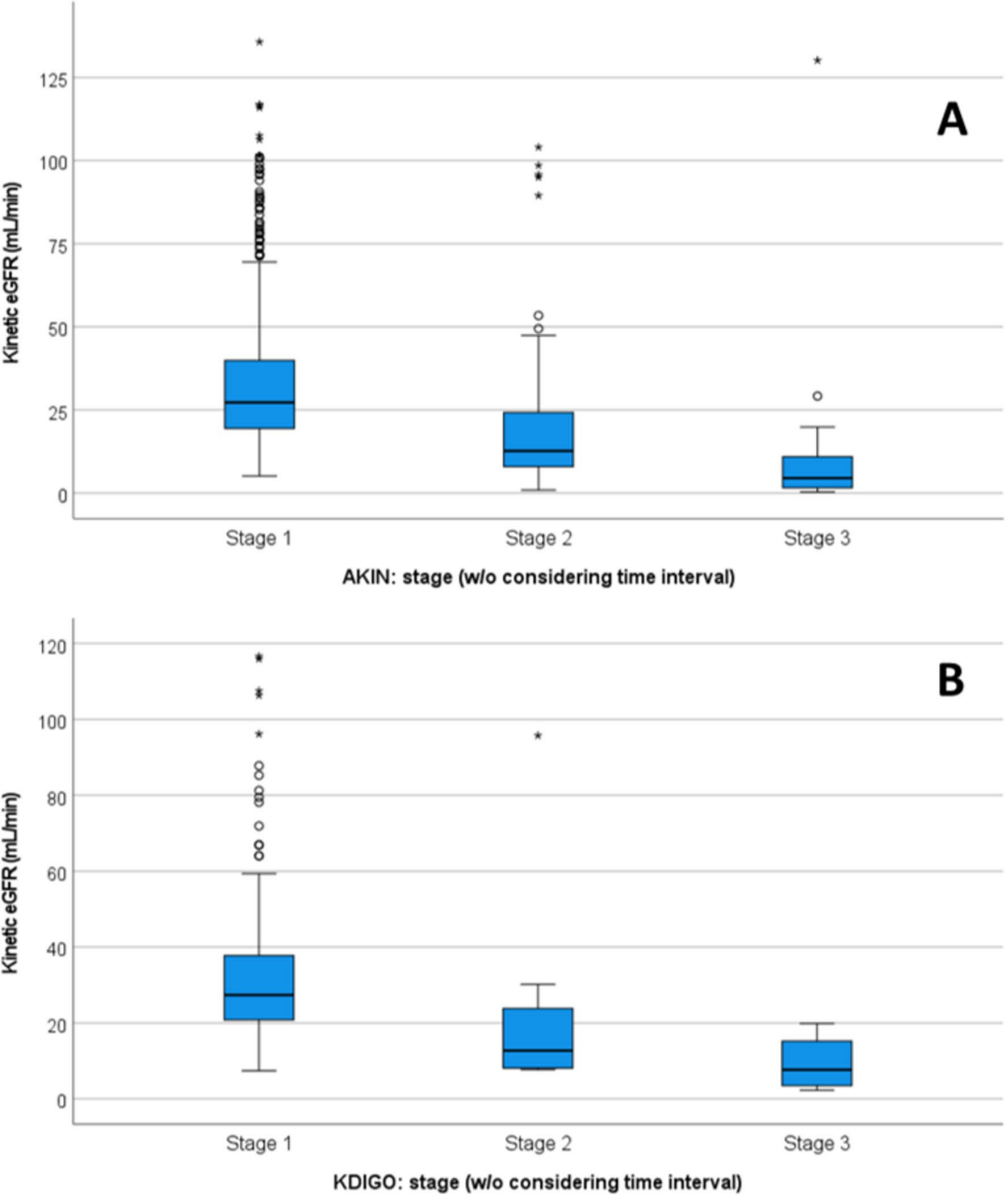
Kinetic estimated glomerular filtration rate (KeGFR) of admissions not identified as with acute kidney injury with AKIN (**A**) and KDIGO (**B**) criteria, but with creatinine elevations over the criteria limits

The lowest eGFR per patient during hospitalization had a median of 61 mL/min/1.73m^2^ (IQR 40:90) when calculated with the CKD-EPI equation). Among the 12,440 hospitalizations where it could be calculated, the lowest KeGFR had a median of 67 mL/min/1.73m^2^ (IQR 43:92). A strong correlation was observed between these two estimates (Spearman’s rho = 0.724; *p* < 0.001). When comparing the KeGFR in the 728 admissions where AKI could not be strictly established using AKIN criteria but had SCr elevations surpassing AKIN limits, a significant difference existed in the KeGFR between different potential AKI stages (Kruskal–Wallis H = 99.582; *p* < 0.001) (Fig. 1A). Similar results were obtained for KDIGO potential AKI stages (Kruskal–Wallis H = 16.999; *p* < 0.001) (Fig. 1B).

Seventy-six drugs, identified in the Renal Drug Handbook (3rd Edition), require dose adjustment in patients with an estimated eGFR of 50 mL/min or lower. In 13,550 hospitalizations (78.5%), patients were prescribed one of these drugs, totalling 88,301 prescriptions. Among the 728 admissions where AKI could not be strictly established using AKIN criteria but had SCr elevations surpassing AKIN limits, 87.9% (640 hospitalizations) involved patients prescribed any of the 76 drugs requiring dose adjustment with eGFR ≤ 50 mL/min, with 556 of these admissions reaching a KeGFR ≤ 50 mL/min/1.73 m^2^. Similarly, in the 370 hospitalizations where AKI could not be strictly established using KDIGO criteria due to the lack of timely SCr assessment, 88.9% (329 hospitalizations) had any of the 76 drugs prescribed, with 300 hospitalizations reaching a KeGFR ≤ 50 mL/min/1.73 m^2^. The impact of considering or ignoring time intervals in establishing AKI using both criteria, combined with the drugs prescribed requiring dose adjustment, is presented in Supplementary File 2.

## Discussion

### Statement of key findings

This study underscores the substantial underdiagnosis of AKI due to the failure to timely measure renal function indicators. Among the 17,269 hospitalizations analysed, only 7.0% and 9.1% could be identified as suffering from AKI strictly using AKIN or KDIGO criteria (considering the time intervals described by those criteria). Notably, in over 46% of hospitalizations, the time between SCr tests exceeded 48 h, raising concerns about the accuracy of strictly applying the time intervals outlined in these criteria. However, in 11.2% of hospitalizations, SCr elevations surpassed the limits described in both criteria, indicating a potential underestimation of AKI. This lack of timely SCr assessment could have resulted in disregarding more than 700 or 370 individuals with AKI (depending on the criteria used), translating to underestimating AKI diagnoses by 60% or 23.5% for AKIN and KDIGO, respectively. This potential AKI underdiagnose was confirmed by the statistical association between the AKI potential stages of these patients with the minimum KeGFR they reached. Also, almost 90% patients suffering unnoticed AKI were using drugs that should have dose adjustment when renal function is impaired.

### Strengths and weaknesses

A key strength of this study lies in its utilization of a real-world cohort of patients, drawing on secondary data obtained for routine patient care. Consequently, the results reflect the current practices of an average Portuguese hospital. The study underscores the imperative for a more standardized protocol to measure inpatient SCr at intervals no longer than 48 h.

The study has limitations. Firstly, it is confined to a single hospital, which lacks representation of all medical specialties, impeding the generalization of results to other European or Portuguese hospitals. The reliance on secondary data extracted from hospital records introduces a potential limitation, as accuracy was not validated on-site. However, the hospital undergoes external accreditation, including a review of medical record data quality. The use of SCr at admission as the steady-state creatinine for KeGFR may introduce inaccuracies, particularly in patients with acute SCr increases just before hospitalization. When analysing exposure to drugs requiring dose adjustment, information on any interventions conducted was unavailable, as such interventions are not recorded. However, this was beyond the scope of our analysis. Lastly, it is essential to note that all four authors are pharmacists by education. While this expertise enriches the interpretation, it is crucial to acknowledge this potential influence when analysing interpretations given.

### Interpretation

This study specifically targeted individuals with normal kidney function at the time of admission, consequently not requiring dose adjustments for renally excreted drugs. However, the failure to timely monitor kidney function might result in overlooking potential AKI occurrences, potentially necessitating drug dose adjustments. Early identification of AKI is critical, as evidence suggests a heightened risk of CKD in patients who have experienced AKI, even in cases where the acute condition completely resolved [22]. Rapid intervention in the earliest stages of AKI could yield long-term benefits for patients [23].

The existing eGFR equations may not accurately reflect glomerular filtration in AKI, as they do in CKD [24]. KDIGO defines AKI based on SCr increases over time and reduced urine output [24]. While various biomarkers have been suggested for measuring filtration rate in AKI [25], their availability and routine use in clinical practice vary. KeGFR has emerged as a valuable alternative, particularly in conditions like AKI where SCr levels change rapidly [14] KeGFR has demonstrated high predictive power for identifying AKI [26] and allows for drug dose adjustments, similar to traditional eGFR equations [27]. Notably, KeGFR could be calculated for nearly 75% of the hospitalizations under analysis.

The management of drug therapy in patients developing AKI remains a topic of debate. Some authors advocate for considering an alternative drug with no dose adjustment requirements in CKD as a first choice [28]. While this option may not always be feasible due to the limited availability of alternative non-renally excreted drugs, it is also not necessarily the most efficient solution as it unnecessarily restricts the therapeutic portfolio. It is crucial to note that the effects of AKI on drugs extend beyond renal excretion, encompassing modifications in hepatic clearance in AKI patients [29]. AKI can increase the volume distribution for hydrophilic drugs due to the shorter half-life of albumin in these patients [30]. Alternatively, some authors advocate for increasing therapeutic drug monitoring in patients with AKI [31]. However, this option may not be universally available for all drugs in all settings. Consequently, drug dose adjustment emerges as the most pragmatic option for many patients developing AKI.

While guidelines for dose adjustment of renally excreted drug in patients with CKD are well established, guidelines in AKI are not so robust. KDIGO recommends increasing research, not only to generate guidelines for “drug dosing adjustments in patients with AKI”, but also to develop electronic tools and decision-making software “to guide drug dosage individualization” [32].

The clinical pharmacist’s role in providing dose adjustment recommendations based on observed modifications in renal function, especially in CKD, is well-documented in the literature of both developed [33, 34] and developing countries [35]. Clinical pharmacists have further demonstrated their contribution to patients recovering from an AKI episode by establishing post-discharge follow-up services [36, 37]. Pharmacists play also a crucial role in AKI prevention by monitoring nephrotoxic drug levels [38], although some studies present conflicting conclusions [39]. Initial results from a large randomized controlled trial revealed that pharmacists, as part of a trained multidisciplinary team, adjusted doses, or initiated drug level monitoring in nearly 70% of patients with AKI [40].

### Further research

Pharmacists can employ intricate dosing techniques to mitigate AKI risks [19] or utilize advanced computerized decision support systems (CDSS) to aid in identifying and preventing iatrogenic risks [40]. However, irrespective of the methods employed, timely access to renal function information is imperative. Pharmacists have advocated for such access and proposed solutions for a considerable duration [41, 42]. In our study, access to SCr data, when available, was provided to hospital pharmacists. Yet, in situations where the SCr test was not conducted promptly, pharmacists should advocate for an expanded scope of practice, a concept already implemented in some countries which entails legal authorization for pharmacists to prescribe specific laboratory tests [43]. Some countries have successfully tested this expanded role, particularly in primary care, involving community pharmacists and general practitioners in the early identification of CKD [44]. However, studies suggest that the location of the clinical pharmacist may influence the delivery and acceptance of interventions [45]. On-ward pharmacists, embedded within multidisciplinary teams, are more likely to have access to all necessary indicators, enhancing the meaningful impact of their interventions [46].

KeGFR emerged as a potentially valuable metric for early AKI identification. However, its calculation, requiring three SCr values (steady state and two within a time interval), may limit its applicability in settings with suboptimal SCr measuring practices. Given the strong correlation observed in our study between KeGFR and eGFR estimated with CKD-EPI, future research should delve into the potential agreement between these two estimates using specialized analytical techniques [47].

## Conclusion

Our study revealed poor real-world SCr testing practices in hospitals for identifying and staging AKI, which could result in a relevant proportion of unnoticed AKI cases. This deficiency may significantly impact medication safety when administering drugs requiring dose adjustment to patients with unknown AKI. To enhance interventions in drug dose adjustment, clinical pharmacists should elevate their monitoring of renal function by ensuring timely assessment of AKI biomarkers.

### Supplementary Information

Below is the link to the electronic supplementary material.

Supplementary file1 (DOCX 68 kb)

Supplementary file2 (DOCX 35 kb)
