# Supplementary material for: Effects of inpatient creatinine testing frequency on acute kidney injury identification and staging: a historical cohort study
Source: Int J Clin Pharm. 2024 Feb 5;46(3):623–30. doi: 10.1007/s11096-023-01697-4 (PMC11133048; doi:10.1007/s11096-023-01697-4)
Supplement: Supplementary file 1 — Supplementary file1 (DOCX 68 kb) [file 11096_2023_1697_MOESM1_ESM.docx]

Appendix 1. Visual representation of the effect of ignoring/not ignoring time intervals in AKI staging


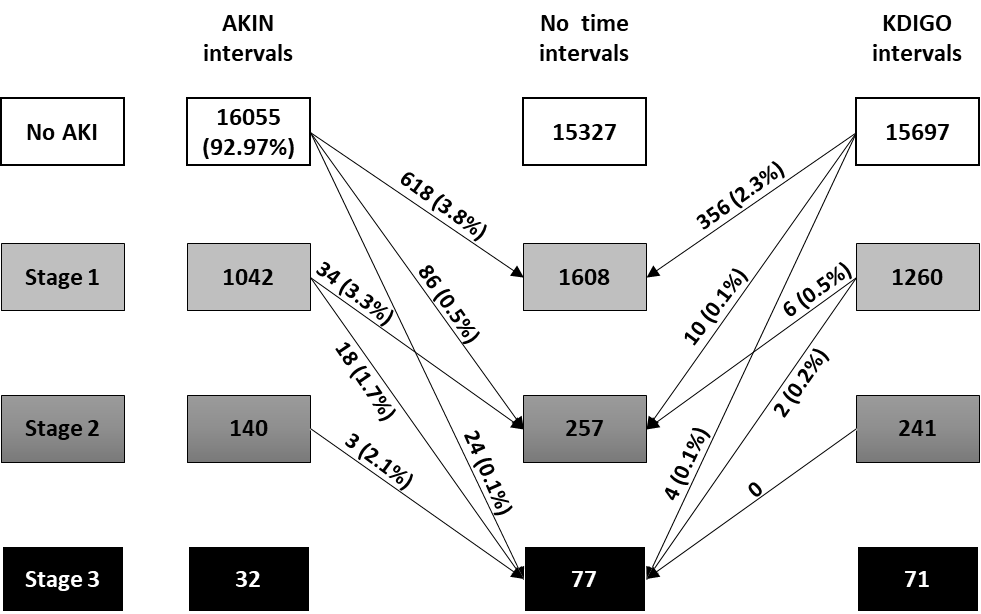


Legend: Number of hospitalizations transitioning AKI stage according to information used on time intervals (% estimated as a function of hospitalizations staged strictly using time intervals in each criterion)
