# Supplementary material for: Effects of inpatient creatinine testing frequency on acute kidney injury identification and staging: a historical cohort study
Source: Int J Clin Pharm. 2024 Feb 5;46(3):623–30. doi: 10.1007/s11096-023-01697-4 (PMC11133048; doi:10.1007/s11096-023-01697-4)
Supplement: Supplementary file 2 — Supplementary file2 (DOCX 35 kb) [file 11096_2023_1697_MOESM2_ESM.docx]

|  | AKIN or KDIGO without considering time intervals | | | | | AKIN: Considering time intervals | | | | KDIGO: Considering time intervals | | | |
| --- | --- | --- | --- | --- | --- | --- | --- | --- | --- | --- | --- | --- | --- |
|  | No AKI | Stage 1 | Stage 2 | Stage 3 | No AKI | | Stage 1 | Stage 2 | Stage 3 | No AKI | Stage 1 | Stage 2 | Stage 3 |
| Any drug with dose adjustment requirement | 9015 (86.1% | 1195 (11.4%) | 207 (2.0) | 57 (0.5%) | 9551 (91.2) | | 786 (7.5) | 113.(1.1) | 24 (0.2) | 9285 (88.6) | 941 (9.0) | 195 (1.9) | 52 (0.2) |
| Acetazolamide (n=18) | 10 (55.6) | 6 (33.3) | 1 (5.6) | 1 (5.6) | 11 (61.1) | | 5 (27.8) | 1 (5.6) | 1 (5.6) | 11 (61.1) | 5 (27.8) | 1 (5.6) | 1 (5.6) |
| Aciclovir IV (n=21) | 13 (61.9) | 7 (33.3) | 1 (4.8) | - | 17 (81.0) | | 4 (19.0) | - | - | 15 (71.4) | 5 (23.8) | 1 (4.8) | - |
| Adefovir (n=0) | - | - | - | - | - | | - | - | - | - | - | - | - |
| Allopurinol (n=430) | 346 (80.5) | 75 (17.4) | 6 (1.4) | 3 (0.7) | 382 (88.8) | | 43 (10.0) | 4 (0.9) | 1 (0.2) | 371 (86.3) | 50 (11.6) | 6 (1.4) | 3 (0.7) |
| Amantadine (n=22) | 20 (90.9) | 2 (9.1) | - | - | 21 (95.5) | | 1 (4.5) | - | - | 20 (90.9) | 2 (9.1) | - | - |
| Amikacin (n=106) | 64 (60.4) | 31 (29.2) | 7 (6.6) | 4 (3.8) | 77 (72.6) | | 23 (21.7) | 4 (3.8) | 2 (1.9) | 73 (68.9) | 25 (23.6) | 5 (4.7) | 3 (2.8) |
| Amisulpiride | 40 (88.9% | 3 (6.7%) | 2 (4.4%) | - | 43 (93.3) | | 3 (6.7) | - | - | 42 (93.3) | 1 (2.2) | 2 (2.4) | - |
| Aspirin (n=2779) | 2401 (91.4) | 211 (7.6) | 23 (0.8) | 4 (0.1) | 2541 (91.4) | | 211 (7.6) | 23 (0.8) | 4 (0.1) | 2482 (89.3) | 248 (8.9) | 39 (1.4) | 10 (0.4) |
| Baclofen (n=80) | 74 (92.5) | 5 (6.3) | 1 (1.3) | - | 79 (98.8) | | - | 1 (1.3) | - | 76 (95.0) | 3 (3.8) | 1 (1.3) | - |
| Bupropion (n=9) | 9 (100) | - | - | - | 9 (100) | | - | - | - | 9 (100) | - | - | - |
| Captopril (n=1344) | 1100 (89.8) | 119 (8.9) | 17 (1.3) | 1 (0.1) | 1207 (89.8) | | 119 (8.9) | 17 (1.3) | 1 (0.1) | 1156 (86.0) | 154 (11.5) | 30 (2.2) | 4 (0.3) |
| Ceftazidime (n=147) | 112 (87.1) | 25 (17.0) | 5 (3.4) | 5 (3.4) | 128 (87.1) | | 16 (10.9) | 1 (0.7) | 2 (1.4) | 116 (78.9) | 22 (15.0) | 5 (3.4) | 4 (2.7) |
| Cefuroxime parent. (n=1498) | 1188 (79.3) | 243 (16.2) | 55 (3.7) | 12 (0.8) | 1301 (86.8) | | 170 (11.3) | 22 (1.5) | 5 (0.3) | 1239 (82.7) | 196 (13.1) | 51 (3.4) | 12 (0.8) |
| Cetuximab (n=0) | - | - | - | - | - | | - | - | - | - | - | - | - |
| Cisplatin (n=2) | 1 (50.0) | 1 (50.0) | - | - | 2 (100) | | - | - | - | 1 (50.0) | 1 (50.0) | - | - |
| Clonazepam (n=332) | 290 (87.3) | 26 (7.8) | 11 83.3) | 5 (1.5) | 307 (92.5) | | 17 (5.1) | 7 (2.1) | 1 (0.3) | 297 (89.5) | 20 (6.0) | 10 (3.0) | 5 (1.5) |
| Clozapine (n=134) | 126 (94.0) | 6 (4.5) | 2 (1.) | - | 130 (97.0) | | 6 (4.5) | 2 (1.5) | - | 128 (95.5) | 4 (3.0) | 2 (1.5) | - |
| Colistin (n=5) | 2 (40.0) | 3 (60.0) | - | - | 2 (40.0) | | 3 (60.0) | - | - | 2 (40.0) | 3 (60.0) | - | - |
| Deferasirox (n=0) | - | - | - | - | - | | - | - | - | - | - | - | - |
| Digoxin (n=682) | 512 (75.1) | 141 (20.7) | 22 (3.2) | 7 (1.0) | 565 (82.8) | | 97 (14.2) | 16 (2.3) | 4 (0.6) | 537 (78.7) | 117 (17.2) | 22 (3.2) | 6 (0.9) |
| Emtricitabine (n=40) | 35 (87.5) | 2 (5.0) | 3 (7.5) | - | 39 (97.5) | | 1 (2.5) | - | - | 37 (92.5) | 1 (2.5) | 2 (5.0) | - |
| Entecavir (n=0) | - | - | - | - | - | | - | - | - | - | - | - | - |
| Enoxaparina (n=9934) | 8534 (85.9%) | 1147 (11.5%) | 199 (2.0%) | 54 (0.5%) | 9043 (91.0) | | 757 (7.6) | 110 (1.1) | 24 (0.2) | 8791 (88.5) | 906 (9.1) | 188 (1.9) | 19 (0.5) |
| Fenofibrato (n=62) | 54 (87.1%) | 8 (12.9) | - | - | 57 (91.9) | | 5 (8.1) | - | - | 56 (90.3) | 6 (9.7) | - | - |
| Gabapentina (n=411) | 352 (85.6) | 47 (11.4) | 10 (2.4) | 2 (0.5) | 376 (91.5) | | 32 (7.8) | 3 (0.7) | - | 364 (88.6) | 36 (8.8) | 9 (2.2) | 2 (0.5) |
| Ganciclovir (n=1) | 1 (100) | - | - | - | 1 (100) | | - | - | - | 1 (100) | - | - | - |
| Gemtamicina (n=308) | 223 (72.4) | 62 (20.1) | 18 (5.8) | 5 (1.6) | 246 (79.9) | | 45 (14.6) | 14 (4.5) | 3 (1.0) | 233 (75.6) | 53 (17.2) | 17 (5.5) | 5 (1.6) |
| Glicazide (m=174) | 147 (93.1) | 11 (6.3) | 1 (0.6) | - | 162 (93.1) | | 11 (6.3) | 1 (0.6) | - | 157 (90.2) | 15 (8.6) | 2 (1.1) | - |
| Hidroxicarbamida (n=35) | 27 (77.1) | 5 (14.3) | 2 (5.7) | 1 (2.9) | 31 (88.6) | | 2 (5.7) | 1 (2.9) | 1 (2.9) | 30 (85.7) | 2 (5.7) | 2 (5.7) | 1 (2.9) |
| Hydroxychloroquine (n=69) | 57 (82.6) | 8 (11.6) | 3 (4.3) | 1 (1.4) | 61 (88.4) | | 4 (5.8) | 3 (4.3) | 1 (1.4) | 60 (87.0) | 5 (7.2) | 3 (4.3) | 1 (1.4) |
| Ibuprofen (n=747) | 703 (94.1) | 35 (4.7) | 8 (1.1) | 1 (0.1) | 720 (96.4) | | 22 (2.9) | 5 (0.7) | - | 711 (95.2) | 28 (3.7) | 8 (1.1) | - |
| Idarubicin (n=0) | - | - | - | - | - | | - | - | - | - | - | - | - |
| Ifosfamide (n=0) | - | - | - | - | - | | - | - | - | - | - | - | - |
| Indometacin (n=24) | 21 (87.5) | 2 (8.3) | 1 (4.2) | - | 22 (91.7) | | 1 (4.2) | 1 (4.2) | - | 22 (91.7) | 1 (4.2) | 1 (4.2) | - |
| Interferon beta (n=0) | - | - | - | - | - | | - | - | - | - | - | - | - |
| Irinotecan (n=0) | - | - | - | - | - | | - | - | - | - | - | - | - |
| Ketorolac (n=1346) | 1232 (91.5) | 93 (6.9) | 20 (1.5) | 1 (0.1) | 1268 (94.2) | | 64 (4.8) | 13 (1.0) | 1 (0.1) | 1246 (92.7) | 79 (5.9) | 18 (1.3) | 1 (0.1) |
| Lamivudine (n=12) | 9 (75.0) | 2 (16.7) | 1 (8.3) | - | 10 (83.3) | | 1 (8.3) | 1 (8.3) | - | 10 (83.3) | 1 (8.3) | 1 (8.3) | - |
| Lamotrigine (n=83) | 73 (88.0) | 10 (12.0) | - | - | 75 (90.4) | | 8 (9.6) | - | - | 73 (88.0) | 10 (12.0) | - | - |
| Levetiracetam (n=577) | 488 (84.6) | 70 (12.1) | 14 (2.4) | 5 (0.9) | 523 (90.6) | | 45 (7.8) | 7 (1.2) | 2 (0.3) | 500 (86.7) | 59 (10.2) | 13 (2.3) | 5 (0.9) |
| Levofloxacin (n=53) | 41 (77.4) | 11 (20.8) | 1 (1.9) | - | 45 (84.9) | | 7 (13.2) | 1 (1.9) | - | 44 (83.0) | 8 (15.1) | 1 (1.9) | - |
| Lisinopril (n=1508) | 1246 (82.6) | 219 (14.5) | 33 (2.2) | 10 (0.7) | 1344 (89.1) | | 149 (9.9) | 12 (0.8) | 3 (0.2) | 1288 (85.4) | 180 (11.) | 30 (2.0) | 10 (0.7) |
| Lithium (n=92) | 87 (94.6) | 5 (5.4) | - | - | 88 (95.7) | | 4 (4.3) | - | - | 88 (95.7) | 4 (4.3) | - | - |
| Mercaptopurine (n=0) | - | - | - | - | - | | - | - | - | - | - | - | - |
| Meropenem (n=159) | 79 (49.7) | 52 (32.7) | 22 (13.8) | 6 (3.8) | 97 (61.0) | | 45 (28.3) | 16 (10.1) | 1 (0.6) | 88 (55.3) | 45 (28.3) | 20 (12.6) | 6 (3.8) |
| Mesalazina (n=80) | 72 (90.0) | 8 (10.0) | - | - | 75 (93.8) | | 5 (6.3) | - | - | 74 (92.5) | 6 (7.5) | - | - |
| Metformin (n=677) | 596 (88.0) | 67 (9.9) | 9 (1.3) | 5 (0.7) | 642 (94.8) | | 35 (5.2) | - | - | 619 (91.4) | 47 (6.9) | 8 (1.2) | 3 (0.4) |
| Methotrexate (n=4) | 4 (100) | - | - | - | 4 (100) | | - | - | - | 4 (100) | - | - | - |
| Morphine (n=1618) | 1149 (71.0) | 337 (20.8) | 99 (6.1) | 33 (2.0) | 1277 (78.9) | | 266 (16.4) | 60 (3.7) | 15 (0.9) | 1194 (73.8) | 295 (18.2) | 96 (5.9) | 33 (2.0) |
| Naproxen (n=135) | 114 (84.4) | 12 (8.9) | 8 (5.9) | 1 (0.7) | 124 (91.9) | | 9 (6.7) | 2 (1.5) | - | 17 (86.7) | 9 (6.7) | 8 (5.9) | 1 (0.7) |
| Neostigmine (n=30) | 8 (26.7) | 13 (43.3) | 7 (23.3) | 2 (6.7) | 10 (33.3) | | 13 (43.3) | 6 (20.0) | 1 (3.3) | 8 (26.7) | 13 (43.3) | 7 (23.3) | 2 (6.7) |
| Nitrofurantoin (n=101) | 82 (81.2) | 15 (14.9) | 2 (2.0) | 2 (2.0) | 91 (90.1) | | 9 (8.9) | 1 (1.0) | - | 85 (84.2) | 13 (12.9) | 1 (1.0) | 2 (2.0) |
| Olanzapine (n=498) | 444 (89.2) | 40 (8.0) | 12 (2.4) | 2 (0.4) | 460 (92.4) | | 33 (6.6) | 4 (0.8) | 1 (0.2) | 449 (90.2) | 37 (7.4) | 10 (2.0) | 2 (0.4) |
| Paliperidone (n=0) | - | - | - | - | - | | - | - | - | - | - | - | - |
| Peginterferoin alfa (n=0) | - | - | - | - | - | | - | - | - | - | - | - | - |
| Piroxicam (n=0) | - | - | - | - | - | | - | - | - | - | - | - | - |
| Pregabalina (n=481) | 414 (86.1) | 53 (11.0) | 12 (2.5) | 2 (0.4) | 441 (91.7) | | 32 (6.7) | 7 (1.5) | 1 (0.2) | 432 (89.8) | 37 (7.7) | 10 (2.1) | 2 (0.4) |
| Quetiapine (n=2532) | 2017 (79.7) | 413 (16.3) | 76 (3.0) | 26 (1.0) | 2225 (87.9) | | 267 (10.5) | 32 (1.3) | 8 (0.3) | 2122 (83.8) | 321 (12.7) | 68 (2.7) | 21 (0.8) |
| Quinine (n=3) | 3 (100) | - | - | - | 3 (100) | | - | - | - | 3 (100) | - | - | - |
| Ribavirine (n=0) | - | - | - | - | - | | - | - | - | - | - | - | - |
| Risperidone (n=563) | 463 (82.2) | 76 (13.5) | 18 (3.2) | 6 (1.1) | 494 (87.7) | | 58 (10.3) | 9 (1.6) | 2 (0.4) | 478 (84.9) | 66 (11.7) | 15 (2.7) | 4 (0.7) |
| Rituximab (n=0) | - | - | - | - | - | | - | - | - | - | - | - | - |
| Rivastigmine (n=60) | 49 (81.7) | 9 (15.0) | 2 (3.3) | - | 53 (88.3) | | 6 (10.0) | 1 (1.7) | - | 50 (83.3) | 8 (13.3) | 2 (3.3) | - |
| Spironolactone (n=1433) | 1052 (73.4) | 323 (22.5) | 46 (3.2) | 12 (0.8) | 1192 (83.2) | | 218 (15.2) | 19 (1.3) | 4 (0.3) | 1135 (79.2) | 246 (17.2) | 42 (2.9) | 10 (0.7) |
| Streptomycin (n=0) | - | - | - | - | - | | - | - | - | - | - | - | - |
| Sucralfate (n=382) | 311 (84.1) | 50 (13.1) | 14 (3.7) | 7 (1.8) | 332 (86.9) | | 40 (10.5) | 6 (1.6) | 4 (1.0) | 320 (83.8) | 41 (10.7) | 14 (3.7) | 7 (1.8) |
| Sulfasalazine (n=17) | 15 (88.2) | 1 (5.9) | 1 (5.9) | - | 16 (94.1) | | 1 (5.9) | - | - | 15 (88.2) | 1 (5.9) | 1 (5.9) | - |
| Tenofovir (n=53) | 46 (86.8) | 3 (5.7) | 3 (5.7) | 1 (1.9) | 50 (94.3) | | 2 (3.8) | - | 1 (1.9) | 48 (90.6) | 2 (3.8) | 2 (3.8) | 1 (1.9) |
| Topotecan (n=0) | - | - | - | - | - | | - | - | - | - | - | - | - |
| Tranexamic ac (n=110) | 71 (64.5) | 29 (26.4) | 9 (8.2) | 1 (0.9) | 81 (73.6) | | 23 (20.9) | 5 (4.) | 1 (0.9) | 74 (67.3) | 26 (23.6) | 9 (8.2) | 1 (0.9) |
| Vangalciclovir (n=2) | 1 (50.0) | 0 | 0 | 1 (50.0) | 1 (50.0) | | 0 | 0 | 1 (50.0) | 1 (50.0) | 0 | 0 | 1 (50.0) |
| Vancomycin (n=207) | 114 (55.1) | 65 (31.4) | 22 (10.6) | 6 (2.9) | 142 (68.6) | | 52 (25.1) | 12 (5.8) | 1 (0.5) | 124 (59.9) | 58 (28.0) | 19 (9.2) | 6 (2.9) |
| Verapamil (n=24) | 18 (75.0) | 4 (16.7) | 2 (8.3) | - | 19 (79.2) | | 4 (16.7) | 1 (4.2) | - | 18 (75.0) | 4 (16.7) | 2 (8.3) | - |
| Vinorelbine (n=0) | - | - | - | - | - | | - | - | - | - | - | - | - |
| Zoledronic ac (n=13) | 9 (69.2) | 2 (15.4) | 2 (15.4) | 0 | 12 892.3) | | 1 (7.7) | 0 | 0 | 10 (76.9) | 2 (15.4) | 1 /7.7) | 0 |
